# Low‐Frequency Stimulation at the Ventromedial Hypothalamus Exhibits Broad‐Spectrum Efficacy Across Models of Epilepsy

**DOI:** 10.1111/cns.70265

**Published:** 2025-02-09

**Authors:** Shuang Zou, Yiwei Gong, Mengqi Yan, Zhijian Yuan, Minjuan Sun, Shuo Zhang, Yuanzhi Yang, Xiongfeng Guo, Lan Huang, Fan Fei, Yi Wang, Zhong Chen, Cenglin Xu

**Affiliations:** ^1^ Zhejiang Key Laboratory of Neuropsychopharmacology, the Second Affiliated Hospital of Zhejiang Chinese Medical University (Zhejiang Xinhua Hospital), School of Pharmaceutical Sciences Zhejiang Chinese Medical University Hangzhou China; ^2^ Department of Pharmacy The First Affiliated Hospital of Zhejiang Chinese Medical University (Zhejiang Provincial Hospital of Chinese Medicine) Hangzhou China

**Keywords:** epilepsy, low‐frequency stimulation, seizure, ventral medial hypothalamus

## Abstract

**Aims:**

The limited efficacy and very restricted antiseizure range of current deep brain stimulation (DBS) targets highlight the need to find an optimal target for managing various seizure types. Here, we aimed to investigate the efficacy of DBS on the ventromedial hypothalamus (VMH) in the different types of experimental epileptic seizures.

**Methods:**

The efficacy of DBS was examined in various epileptic seizure models, and the potential mechanisms were investigated by using in vivo calcium signal recording and optogenetics.

**Results:**

The *c‐fos* expression was significantly increased in the glutamatergic neurons of VMH (VMH^glu^) following seizures. Then, 1‐Hz low‐frequency stimulation (LFS) at the VMH successfully attenuated the seizure severities across models of epilepsy, including the maximal electroshock, the pentylenetetrazol, the absence seizure, the cortical or hippocampal kainic acid–induced acute seizure, and the hippocampal‐kindling models. The in vivo calcium imaging recordings revealed that LFS could inhibit the activities of the VMH^glu^. Optogenetic inhibition of VMH^glu^ mirrored LFS's antiseizure impact. Further anterograde viral tracing confirmed the extensive distributed projections of VMH^glu^, which may compose the circuitry basis of the broad‐spectral efficacy of LFS.

**Conclusion:**

These findings demonstrate that VMH‐LFS is a broad‐spectrum treatment approach for different seizure types by decreasing VMH^glu^ activity.

## Graphical abstract

Low‐frequency stimulation (LFS) at VMH alleviated seizure severities across models. VMH LFS also retarded the epileptogenesis in the hippocampal‐kindling model. LFS attenuated the activation of the glutamatergic neurons in VMH during seizures.

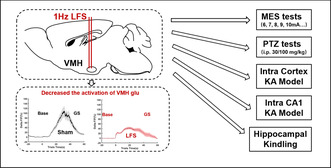

## 1 Introduction

Given the high incidence of being intractable (nearly one‐third), pharmacoresistant epilepsy, which was defined as the failure of adequate trials of two tolerated and appropriately chosen and used antiseizure medication schedules to achieve sustained seizure freedom, emerges as one of the most challenging conditions for neurologists [1]. Deep brain stimulation (DBS) has been accepted for controlling seizures of intractable epilepsy [2, 3]. Current evidence suggests that the anterior thalamic nucleus (ATN), centromedian thalamic nucleus (CMT), and hippocampus are suitable targets for DBS in epilepsy [4, 5, 6]. However, DBS at each therapeutically feasible target appears to prioritize various seizure types and seizure origin zones [7, 8]. Detailly, the efficacy of CMT‐DBS was restricted in generalized‐onset seizures [7, 9], while focal‐onset seizures originating from the temporal and frontal lobes were responsive to ATN‐DBS [8, 9], hippocampal DBS was mainly chosen for temporal lobe epilepsy [10]. None of these current targets could provide broad‐spectrum efficacy across seizure types. And this shortage may underlie the fact that DBS merely reaches an average effective rate of nearly 50%–60% [8, 9, 10, 11]. Thus, searching for a new effective DBS target for diverse seizure types may be a vital step toward meeting this critical clinical criterion.

The hypothalamus has garnered significant attention in epilepsy studies [12, 13]. Seizures originating in different areas would propagate to the hypothalamus via dense projections, potentially leading to chaos in hypothalamic functioning. Also, hypothalamic–pituitary–gonadal (HPG) axis anomalies are frequently identified in epilepsy, and resultant gonadal hormone activities are hypothesized to aggravate or ease the epileptic conditions [12, 14, 15]. Besides epileptic conditions, a hyperactive hypothalamic–pituitary–adrenal (HPA) axis and its neuroanatomic and neuropathologic complications, as well as disturbances in serotonergic, noradrenergic, γ‐aminobutyric acid (GABA)ergic, and glutamatergic neurotransmitter systems, were also involved in epileptic comorbidities [16]. The ventromedial hypothalamus (VMH) is recognized as a critical region with a variety of physiological functions [17, 18, 19]. Earlier limited literatures have confirmed the drastically enhanced *c‐fos* expression in VMH, regardless of seizure origin or types [20, 21]. Furthermore, the reproductive endocrine problem, which is a prevalent comorbidity in epilepsy, is hypothesized to be linked to the altered function of VMH [12]. These findings show that VMH may be a significant region involved in several sorts of seizure activities, and whether it is a suitable target for broad‐spectral seizure control needs additional exploration. Here, through testing the efficacy of VMH‐DBS across models of epilepsy, we provided the first experimental evidence that low‐frequency stimulation (LFS) at the VMH exhibits a broad‐spectral antiseizure efficacy, which possesses potential translational significance.

## 2 Materials and Methods

### 2.1 Animals

The male C57/6 J mice (WT, RRID: IMSR_JAX:000664, SLAC Laboratory Animal Centre) and *vGlut2‐Cre* mice (The Jackson Laboratories, stock number: 016963; mutant reverse sequence 5′ → 3′: ACA CCG GCC TTA TTC CAA G, common sequence 5′ → 3′: AAG AAG GTG CGC AAG ACG, and wild‐type reverse sequence 5′ → 3′: CTG CCA CAG ATT GCA CTT GA) were used. All animals (25–30 g, 10 ~ 12 weeks old) were housed in ventilated cages and kept under a 12‐h light/dark cycle. All the experiments were approved by the guidelines of the Animal Advisory Committee of Zhejiang Chinese Medical University (Approved Number: 20230306–10). The care and use of animals were in complete compliance with the Animal Research: Reporting of In Vivo Experiments (ARRIVE) guidelines.

### 2.2 Virus

Cre‐inducible recombinant adeno‐associated virus (AAV) vector containing GCaMP6m (*pAAV‐EF1a‐DIO‐GCaMP6m‐WPRE*, serotype: AAV2/9, viral titers: 9.78 × 1012 vg/mL, 200 nL) or *pAAV‐CAG‐FLEX‐ArchT‐GFP* (serotype: AAV2/9, viral titers: 1.75 × 1013 vg/mL, 200 nL) was injected into the VMH of *Vglut2‐Cre* mice for fluorometric monitoring or optogenetic manipulation of VMH glutamatergic neurons, respectively. All viruses were from the Brain VTA Co. Ltd.

### 2.3 Stereotactic Implantation

Mice were firstly anesthetized with sodium pentobarbital (i.p., 60 mg/kg) and then mounted in a stereotaxic apparatus (RWD). In all the mice, bipolar electrodes (each 0.125 mm in diameter; A.M. Systems, USA) were implanted into the right VMH (AP: −1.4 mm, L: −0.4 mm, and V: −5.6 mm). In the KA models, a custom‐made cannula electrode was simultaneously implanted into the ipsilateral right primary motor cortex M1 (AP: 1.0 mm; L: −1.5 mm; and V: −1.5 mm) or right hippocampal CA1 (AP: −1.6 mm; L: −2.0 mm; and V: −2.0 mm) [22, 23]. In the kindling model, bipolar electrodes were implanted into the ipsilateral right hippocampal CA3 (AP: −2.9 mm; L: −3.2 mm; and V: −3.2 mm) [24]. In the PTZ model, two screws were placed over the cortex to record EEG. All the behavioral experiments were performed 1 week after surgery. The coordinates were measured according to the standard mouse brain atlas [25]. Only mice with correct locations of electrodes and cannulas, along with viral expression, were included in the results.

### 2.4 The Pentylenetetrazol Models

PTZ (i.p., 100 or 30 mg/kg) was *i.p*. injected to elicit the acute generalized onset seizures [26, 27]. Then, behavioral observations and EEG recordings (PowerLab system, AD Instruments) were performed for 30 min. In 100 mg/kg PTZ‐treated mice, seizure severities were defined according to a revised Racine's score, as follows: Stage 1, whisker trembling during behavioral arrest; Stage 2, facial jerking expressed with the nose; Stage 3, neck jerks; Stage 4, clonic seizures while the animal falls into a sitting position; Stage 5, tonic–clonic seizures with the animal falling on its side; and Stage 6, wild jumping and tonic extension [28]. Stage 5 and 6 seizures were regarded as typical generalized myoclonic seizures. In 30 mg/kg PTZ‐treated mice, typical absence seizure was objectively quantitated by measuring the numbers and durations of the bilaterally synchronous spike‐wave discharge (SWD) during the 30‐min observation period [29]. Typical SWD was defined as repetitive spikes (with a frequency of 5–8 Hz) which is threefold higher in amplitude compared to the baseline [30]. The duration of each SWD was calculated from the beginning of the first spike to the end of the last one.

### 2.5 The Maximal Electroshock Model

The MES model was established according to our previous study [31]. Briefly, MES stimulations (50 Hz, duration 0.2 s) were delivered by a rodent shocker (Hugo Sachs Elektronik, March‐Hugstetten, Germany) through saline‐moistened auricle electrodes (HSE‐HA, Hugo Sachs Elektronik, Freiburg, Germany). The initial stimulus current intensity was 7 mA and raised at 1 mA per minute. The threshold of MES was defined by the minimum current intensity at which the seizure severity reaches generalized‐onset tonic–clonic seizures (Stage 3 seizures characterized by tonic hind limb extension at a 180° angle to the torso).

### 2.6 The Kainic Acid Models

The KA‐induced focal‐onset seizure models were established according to our previous studies [22, 23]. Briefly, KA (ab120100, Abcam, 0.25 μg in 0.5 μL saline) was injected into the right CA3 or M1 cortex using an infusion needle (RWD). The injection was performed by a microsyringe (Hamilton) at a rate of 0.2 μL/min. EEGs in the hippocampus or the M1 cortex were recorded in the subsequent 1.5 h by using a PowerLab System (AD Instruments) at a sampling rate of 1 kHz. Seizure severities were according to the Racine's scale: (1) facial movement; (2) head nodding; (3) unilateral forelimb clonus; (4) bilateral forelimb clonus and rearing; and (5) rearing and falling [32]. Stage 4–5 seizures were regarded as generalized seizures.

### 2.7 The Hippocampal‐Kindling Model

The hippocampal‐kindling model was conducted according to previous study [24]. The after‐discharge threshold (ADT) was determined (monophasic square‐wave pulses, 20 Hz, 1 ms/pulse, 40 pulses) by a constant current stimulator (AM‐1000, A.M. System), and the EEG was recorded by a Neuronscan amplifier. The stimulation intensity began at 40 μA and increased by 20 μA in each step with an interval of 1 min. The minimal intensity that could elicit ≥ 5 s after discharge was deemed as the ADT. Then daily 10 kindling stimulations (monophasic square‐wave pulses, 400 μA, 20 Hz, 1 ms/pulse, 40 pulses, and 30 min interval) were applied. Seizure severity was evaluated by seizure stage and after‐discharge duration (ADD). The seizure stages were classified according to Racine's scale [32]. Mice that exhibited three consecutive Stage 5 seizures were regarded as fully kindled.

### 2.8 DBS

Here, DBS was delivered via the previously implanted bipolar electrodes in the VMH. In brief, monophasic square‐wave pulses with a width of 0.1 ms were applied, and different frequencies of 1, 30, and 100 Hz were selected. In the MES model, DBS was delivered 10 min prior to the MES tests and kept through the whole experimental process (Figure 2A). In the PTZ models, continuous DBS was delivered immediately after PTZ injection for 30 min (Figure 2F,M). In both cortical and hippocampal KA models, DBS was applied immediately after KA injection and lasted for 90 min (Figure 3B,I). In the hippocampal‐kindling model, 5‐min‐long DBS was given after each kindling stimulation (Figure 4B,L).

### 2.9 Optical Fiber Photometry and Optogenetic Manipulation

The optic fiber photometry system (Thinkertech) includes a 488 nm diode laser (OPIS 488LS; Coherent), a dichroic mirror (MD498, Thorlabs), an optic fiber (0.23 mm Core, 0.37 NA, Inper) coupled with an ×10 lens (Olympus), and fiber launch (Thorlabs). The fluorescence GCaMP6 was bandpass filtered and collected by a photomultiplier tube and converted into the analog voltage signals (sample rate 100 Hz). The voltage signals were further analyzed in MATLAB. The fluorescence changes (*ΔF/F*) were calculated with the following equation: (*ΔF/F*) = (F − F0)/F0 [23].

For optogenetic manipulation, direct yellow light (589 nm) was delivered for 5 min via a 200‐μm‐diameter optical fiber (0.23 mm Core, 0.37 NA, Inper), which was controlled by a Master‐8 System (AMPI, Israel) for inhibition.

### 2.10 Immunohistochemistry

Deep anesthetized mice were intracardially perfused with 0.9% saline and freshly prepared using 4% paraformaldehyde (PFA). For *c‐fos* staining, mice were sacrificed 1.5 h after behavioral tests. Dehydrated brains were cut into slices (25 μm) with a freezing microtome (Thermo Scientific). Brain slices were washed three times with PBS, incubated in a blocker solution (1% bovine serum albumin and 3% Triton X‐100 in 0.05 M PBS) containing 5% donkey serum, and kept for 2 h at room temperature. After that, the primary antibody anti‐*c‐fos* (1:1000, ab208942, mouse monoclonal, Abcam) was incubated in a 4°C refrigerator overnight for *c‐fos* staining only. For double‐staining experiments, antibody anti‐Vglut2 (1:100, TA322266S, rabbit polyclonal, OriGene) was incubated along with the anti‐*c‐fos* (1:1000). On the next day, slices were incubated with the secondary conjugated antibody (Alexa Fluor 488, donkey anti‐mouse IgG H&L, 1:1000, ab150105, Abcam), then washed and mounted with DAPI (Vectashield Mounting Media, Vector Labs). Images were captured by confocal microscopy (SP8, Leica). Images were analyzed in ImageJ software (1.52a version).

### 2.11 Statistics

Statistical analyses were performed using GraphPad Prism software (Version 9.1.1). All data were presented as the mean ± S.E.M. The number of experimental replicates (*n*) is indicated in the Figure legend and refers to the number of experimental subjects in each experimental condition. Shapiro–Wilk normality test was used to evaluate the normality of each dataset. The appropriate statistical methods were indicated in the Figure legend, respectively.

## 3 Results

### 3.1 The Glutamatergic Neurons in the VMH Were Activated After Seizures

**FIGURE 1:**
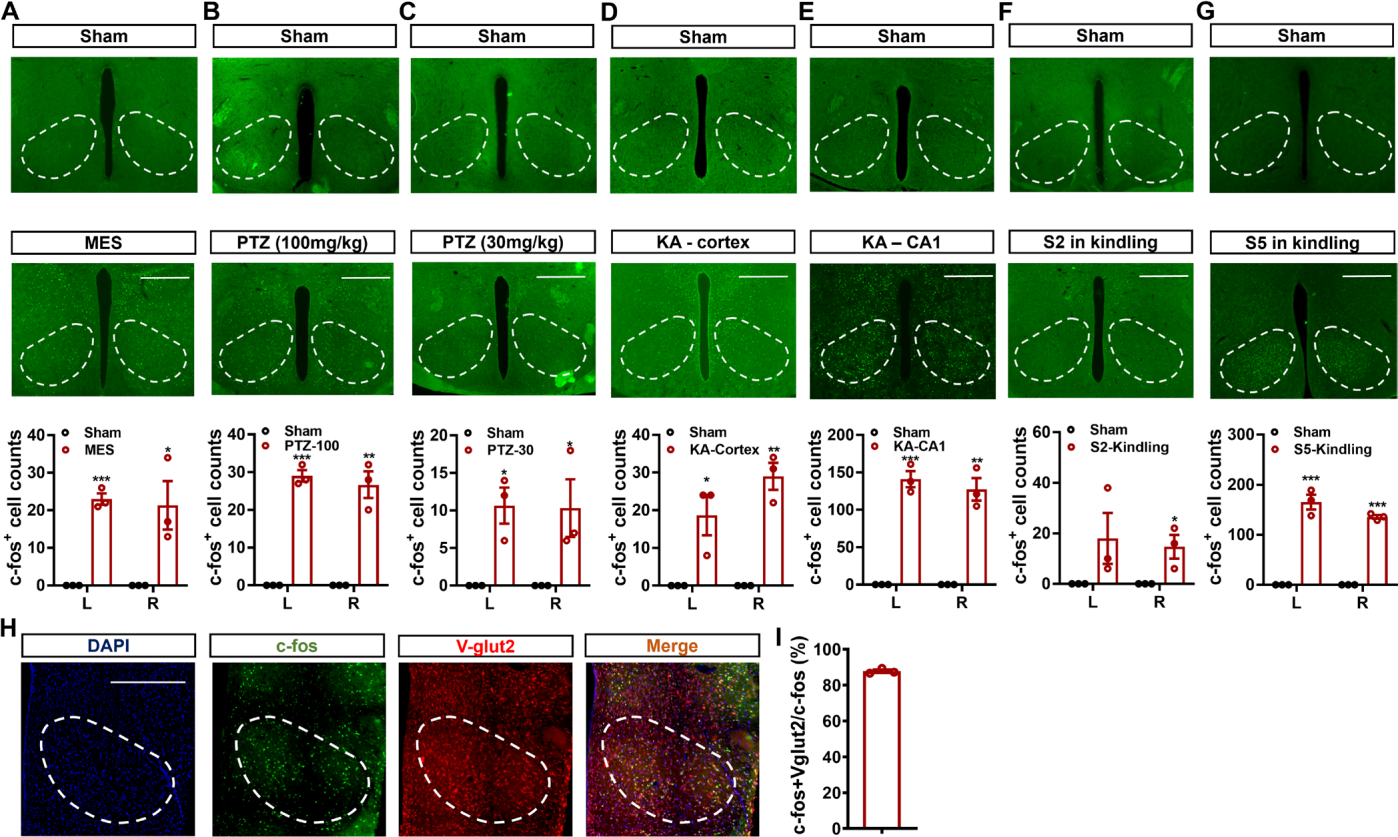
Glutamatergic neurons in the ventromedial hypothalamus were activated across models of epilepsy. The numbers of *c‐fos*‐positive cells in the VMH of mice experiencing the MES‐induced acute seizures (A), the 100 or 30 mg/kg PTZ‐induced acute seizures (B and C), the KA‐induced acute seizures (D and E), or the hippocampal‐kindling–induced stage 2 and 5 seizures (F and G) were compared with corresponding sham conditions (*n* = 3 for each experiment). (H) The representative costaining image of *c‐fos* and Vglut2 in the VMH of mice experienced hippocampal‐kindling–induced stage 5 seizure. (I) The coexpression rate of *c‐fos* and Vglut2 in the VMH (*n* = 3). *p < 0.05, **p < 0.01, ***p < 0.001, unpaired *t* test; L: the left hemisphere, R: the right hemisphere.

Initially, we wanted to investigate the neural responses to seizure activities in the bilateral VMH. Through performing *c‐fos* staining, it can be observed that compared with the sham group, the numbers of *c‐fos*‐positive signals were significantly higher in mice that experienced seizures caused by the MES tests (Figure 1A), the PTZ injection (both 100 and 30 mg/kg, Figure 1B,C), the cortical or hippocampal KA injection (Figure 1D,E), and the hippocampal kindling (Figure 1F for Stage 2 seizures and Figure 1G for Stage 5 seizures). Specifically, we discovered that the quantities of c‐fos‐positive signals were comparable between the right and left VMH, showing that neurons in the VMH were significantly engaged during seizures across epilepsy models. Given that the neurons in the VMH are primarily glutamatergic, we double‐stained *c‐fos* and *V‐glut2* (a common marker for glutamatergic neurons) in hippocampal‐kindled mice. As revealed in Figure 1H, approximately 90% of *c‐fos*‐positive neurons co‐stained with *Vglut2* (Figure 1I). Taken together, these findings revealed that glutamatergic neurons in the VMH (VMH^glu^) were significantly activated during different types of seizures.

### 3.2 VMH‐LFS Alleviated Seizure Severities in Acute Generalized Seizure Models

**FIGURE 2:**
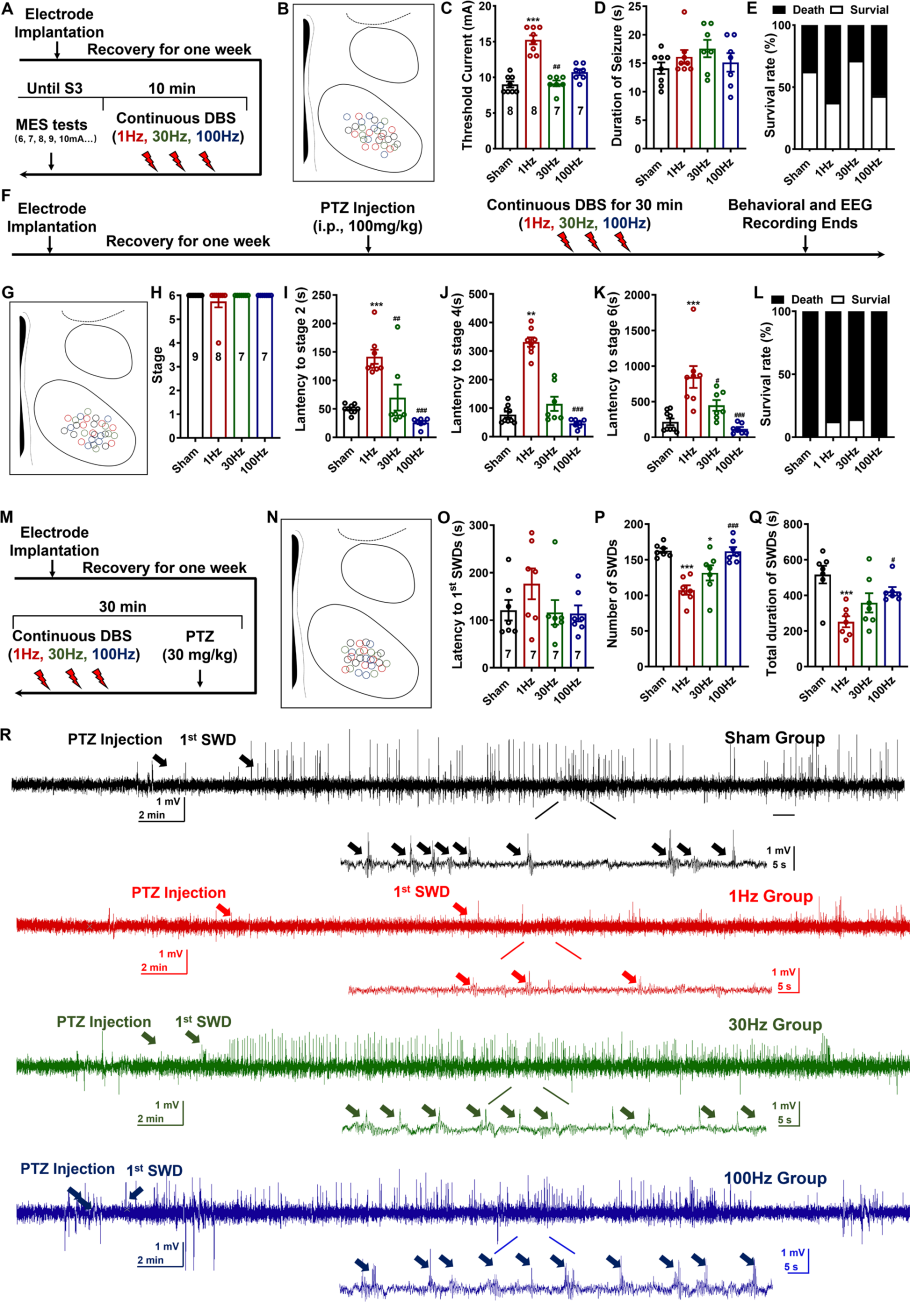
Low‐frequency stimulation at the ventromedial hypothalamus protects against generalized‐onset seizures. (A) The schematic of the MES experiments. (B) All the electrode traces in the VMH of the MES experiments (black: sham group, red: 1 Hz group, green: 30 Hz group, and blue: 100 Hz group). (C) The comparisons of the threshold currents to elicit generalized‐onset tonic–clonic seizures among all the groups. (D) The durations of generalized‐onset tonic–clonic seizures in each group. (E) The survival rates of mice experiencing the MES tests. (F) The experimental process to test the efficacy of VMH‐DBS on PTZ‐induced generalized‐onset myoclonic seizures. (G) The summary of the electrode traces in the VMH. (H–K) The efficacy of VMH‐DBS for the highest seizure stages (H), the latencies to the occurrence of the Stage 2 seizures (I), the Stage 4 seizures (J), and the Stage 6 seizures (K). (L) The survival rates of mice experienced 100 mg/kg PTZ injection. (M) The schematic of the 30 mg/kg PTZ experiment process. (N) The distribution of the electrodes in the VMH. (O–Q) The effects of VMH‐DBS on the latency to the occurrence of first SWDs (O), the total numbers of SWDs (P), and the total duration of the SWDs (Q). (R) The representative seizure EEGs and spectrums in the 30 mg/kg PTZ‐induced acute seizure models. The seizure EEGs and spectrums of the sham (black), 1 Hz (red), 30 Hz (green), and 100 Hz (blue) groups were shown. The arrows denoted the PTZ injection and the representative SWDs. For the MES experiments, *n* = 8 for both the sham and 1 Hz groups, and *n* = 7 for both the 30 and 100 Hz groups. In the 100 mg/kg PTZ experiments, *n* = 9 for the sham group, *n* = 8 for the 1 Hz group, and *n* = 7 for both the 30 and 100 Hz groups. In the 30 mg/kg PTZ experiments, *n* = 7 for all the groups. *p < 0.05, ***p < 0.001, compared with the sham group; ^#^ *p* < 0.05, ^##^ *p* < 0.01, ^###^ *p* < 0.001, compared with the 1 Hz group; nonparametric Kruskal–Wallis test was used for C and J, one‐way ANOVA followed by Turkey's post hoc test was used for other comparisons.

According to the latest guidelines proposed by the International League Against Epilepsy, seizures can be mainly divided into generalized and focal‐onset types [33]. Then, we wanted to see how effective VMH‐DBS was in the MES model, which mimicked typical generalized‐onset tonic–clonic seizures [34]. The experimental techniques are depicted in Figure 2A. All the electrodes in this experiment were strictly positioned at the VMH (Figure 2B). Interestingly, we discovered that 1 Hz LFS increased threshold currents relative to the sham group, although neither 30 nor 100 Hz DBS was effective (Figure 2C). However, VMH‐DBS had no effect on the duration of tonic–clonic seizures or survival rates (Figure 2D,E).

The efficacy of 1 Hz VMH‐LFS against PTZ‐induced (100 mg/kg) generalized‐onset myoclonic seizures was then investigated (Figure 2F). Figure 2G shows the postexamination of electrode placement. Behavioral data revealed that 1 Hz LFS effectively reduced seizure severity in the PTZ paradigm, as seen by longer latencies to Stage 2, 4, and 6 seizures (Figure 2H–L). Like the MES model, the efficacy was limited to 1 Hz LFS; neither 30 nor 100 Hz DBS showed such effects (Figure 2H–L). The representative EEGs and spectrums are shown in Figure S1.

The aforementioned results confirmed that VMH‐LFS was effective for both generalized‐onset tonic–clonic and myoclonic seizures. Thus, whether VMH‐DBS could be effective for generalized‐onset absence seizures was further examined by injecting a lower dose (30 mg/kg) of PTZ into the mice (Figure 2M,N). As shown in Figures 1, 2O–Q Hz LFS exhibited a tendency to prolong the latency to the appearance of occurrence of first SWD and effectively reduced the numbers of SWDs, as well as shortened the SWD durations. Interestingly, 30 Hz DBS also exhibited a weaker effect on the number of SWDs (Figure 2P). The representative EEGs and spectrums are shown in Figure 2R.

### 3.3 VMH‐LFS Protects Against Acute Focal‐Onset Seizures in KA Models

**FIGURE 3:**
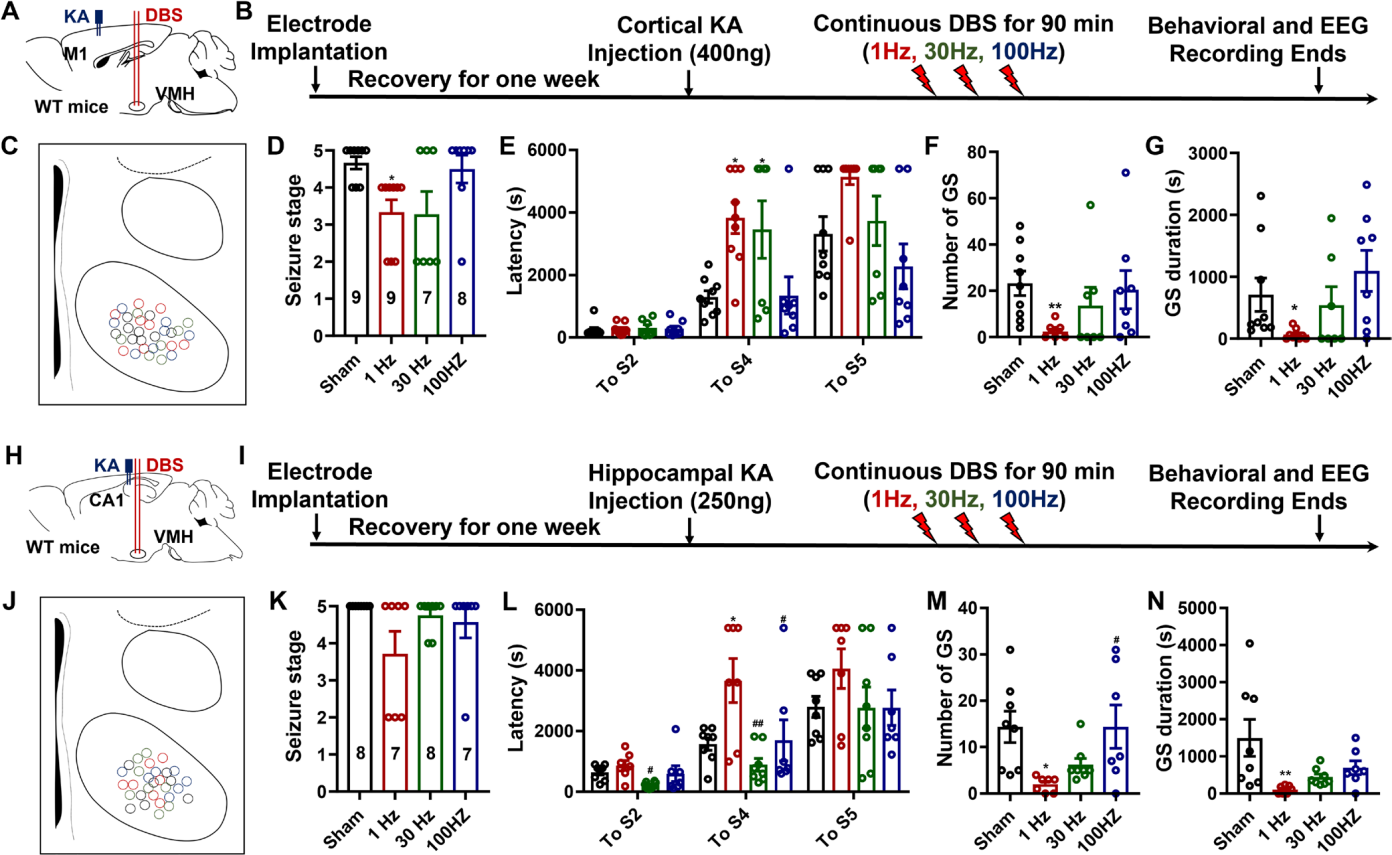
The efficacy of low‐frequency stimulation at the ventromedial hypothalamus in the KA models. (A) The schematic of the electrodes and cannula locations in the cortical KA model. (B) The experimental design, after the cortical KA injection, VMH‐DBS was delivered, and the behavioral and EEG recordings were performed for 90 min. (C) The distributions of the electrodes in the VMH. (D–G) The efficacy of VMH‐DBS on the highest seizure stages (D), the latencies to Stage 2, 4, and 5 seizures, the number of GS (F), and the total duration of the GS (G). (H) The schematic of the electrodes and cannula locations in the hippocampal KA model. (I) The schematic of the experimental process, VMH‐DBS was delivered after the hippocampal KA injection, and the behavioral and EEG recordings were performed for 90 min. (J) The distributions of the electrodes in the VMH. (K–N) The efficacy of VMH‐DBS on the highest seizure stages (K), the latencies to Stage 2, 4, and 5 seizures (L), the number of GS (M), and the total duration of the GS (N). In the cortical KA model, for both the sham and 1 Hz groups, *n* = 9; *n* = 7 or 8 for the 30 and 100 Hz groups, respectively. In the hippocampal KA model, for both the sham and 30 Hz groups, *n* = 8; for the 1 and 100 Hz groups, *n* = 7. *p < 0.05, **p < 0.01, compared with the sham group; ^#^ *p* < 0.05, ^##^ *p* < 0.01, compared with the 1 Hz group. Nonparametric Kruskal–Wallis test was used for D, F, G, and K. One‐way ANOVA followed by Turkey's post hoc test was used for other comparisons.

Unlike generalized‐onset seizures, which originate in both hemispheres, focal‐onset seizures begin in one area of the brain and spread to others [33]. Thus, we created acute focal‐onset seizure models by injecting KA intracerebrally into the motor cortices or the hippocampus CA1. The experimental procedure and electrodes' location in the cortical KA models are shown in Figure 3A–C. As denoted in Figure 3D, both 1 and 30 Hz DBS effectively reduced the highest seizure stages caused by cortical KA injection. Correspondingly, mice receiving 1 or 30 Hz DBS exhibited prolonged latencies to Stage 4 seizures (Figure 3E). However, 1 Hz LFS appeared to be effective only on the number and duration of secondary generalized seizures (GS, Figure 3F,G). The representative seizure EEGs and spectrums are denoted in Figure S2.

Similarly, we investigated the effectiveness of VMH‐DBS in the hippocampal KA model (Figure 3H–J). Unlike the cortical KA model, merely LFS may inhibit the highest seizure stages (Figure 3K), prolong the latencies to S4 seizures (Figure 3L), and reduce both the numbers and total durations of GS (Figure 3M,N). While neither 30 nor 100 Hz VMH‐DBS could effectively treat seizures generated by hippocampus KA injection, as seen in the representative seizure EEGs and spectrums (Figure S3). To summarize, VMH‐LFS was effective against focal‐onset seizures, particularly focal‐onset secondary generalized seizures, in both cortical and hippocampal KA models.

### 3.4 VMH‐LFS was Both Antiepileptogenesis and Anticonvulsant in the Hippocampal‐Kindling Model

**FIGURE 4:**
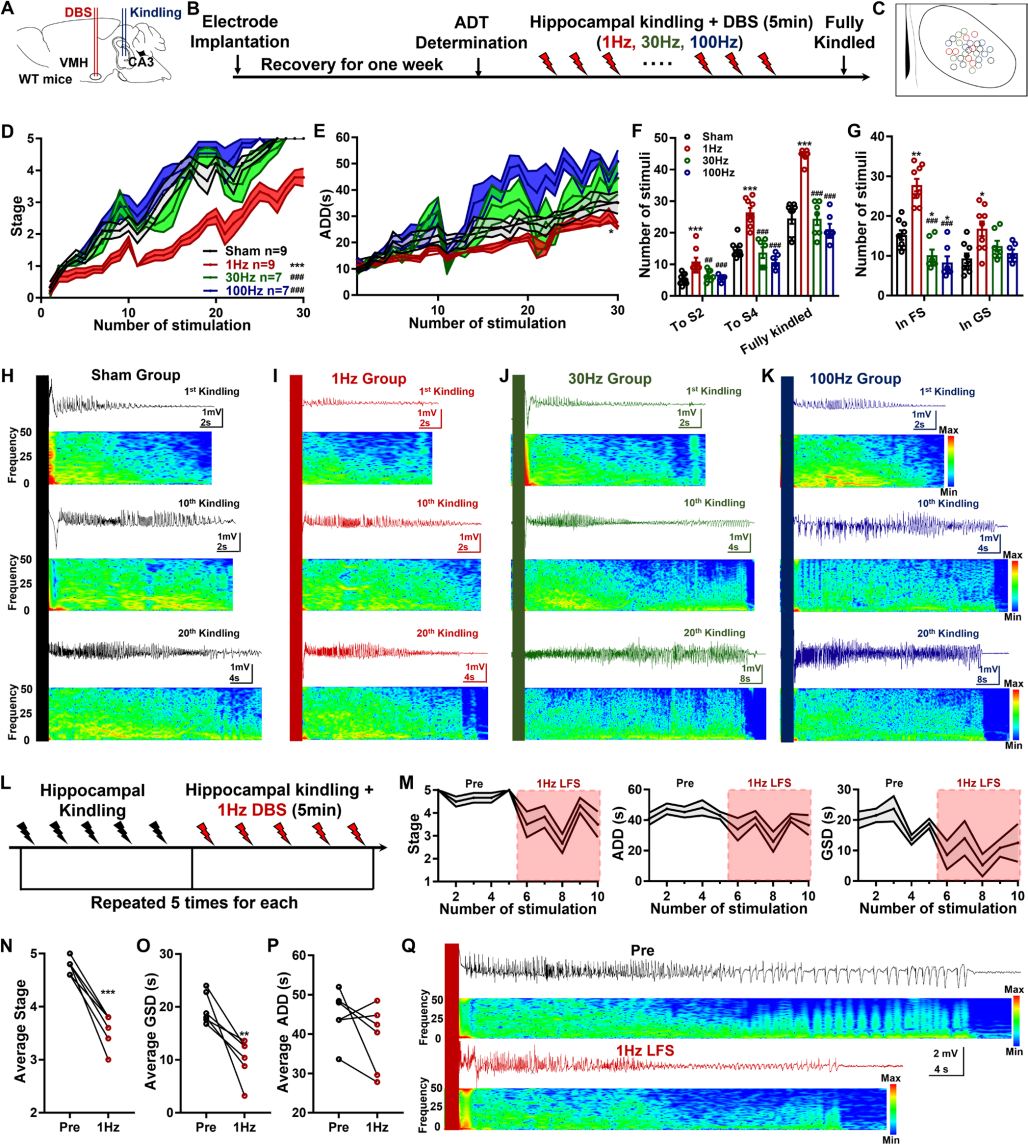
Low‐frequency stimulation at the ventromedial hypothalamus retarded the epileptogenesis process and inhibited fully kindled generalized seizures in the hippocampal‐kindling model. (A) The electrodes were planted into the ventral CA3 and the VMH. (B) The schematic of the experimental design for the hippocampal‐kindling model. (C) The electrode distribution in the VMH. (D and E) The progression of seizure stages (D) and ADDs (E) in the hippocampal‐kindling model. (F) The number of kindling stimulations needed to reach Stages 2 and 4, and fully kindled seizures. (G) The number of kindling stimulations that mice experienced to stay in FS (Stages 0–3) and GS (Stages 4 and 5). (H‐K) The representative seizure EEGs and spectrums when receiving 1st, 10th, and 20th kindling stimulation for each group. (L) The schematic of the experimental design for testing the efficacy of VMH‐LFS on the fully kindled seizures. (M) The progression of seizure stages, ADDs, and GSDs before and during VMH‐LFS. (N–P) The comparison of the averaged seizure stages (N), GSDs (O), and ADDs (P) before and during VMH‐LFS. (Q) The representative seizure EEGs and spectrums in fully kindled mice before and during VMH‐LFS. For the kindling process, *n* = 9 for both the sham and 1 Hz groups, and *n* = 7 for both 30 and 100 Hz groups. For D and E, *p < 0.05, ***p < 0.001, compared with the sham group; ^###^ *p* < 0.001, compared with the 1 Hz group, two‐way ANOVA. For F and G, *p < 0.05, **p < 0.01, ***p < 0.001, compared with the sham group; ^##^ *p* < 0.01, ^###^ *p* < 0.001, compared with the 1 Hz group; one‐way ANOVA followed by Turkey's post hoc test. For fully kindled mice, a total of six mice were included. For M and O, **p < 0.01, ***p < 0.001, compared with the preconditions, paired *t* test.

Given that VMH‐LFS was effective for both acute generalized and focal‐onset seizures, we wanted to investigate its efficacy in the hippocampus‐kindling paradigm, which may mirror the chronic epileptogenesis process (Figure 4A–C). As shown in Figure 4D, compared with the sham group, mice receiving LFS exhibited a significantly retarded progression of seizure stages and ADDs, while 30 and 100 Hz VMH‐DBS showed no such effects (Figure 4D,E). These phenomena were further reflected in the number of stimuli needed to reach or stay in different seizure stages. As shown in Figure 4F,G, mice in the 1 Hz LFS group possessed more numbers of stimulations needed to reach Stages 2 and 4 and fully kindled seizures, as well as more numbers of stimulations to stay in FS (Stages 0–3) and GS (Stages 4 and 5). The antiepileptogenesis efficacy of 1 Hz LFS was further reflected in the representative seizure EEGs and spectrums (Figure 4H–K).

Then, whether LFS could also alleviate the severities of fully kindled seizures (mimics of typical focal‐onset secondary generalized seizures) in the hippocampal‐kindling model was examined (Figure 4L). We found that fully kindled mice would exhibit stable Stage 4 or 5 seizures at the baseline condition, while LFS had a reduced impact on the seizure stages and generalized seizure durations on fully kindled mice (GSD, Figure 4M). Through performing statistical comparisons, we confirmed that VMH‐LFS effectively reduced the average seizure stages and shortened the average GSDs, which were reflected in the representative seizure EEGs and spectrums (Figure 4N–Q). Thus, these results indicated that VMH‐LFS was antiepileptogenesis and anticonvulsant in the hippocampal‐kindling model.

### 3.5 Neuronal Inhibition was Associated With the Effects of LFS

**FIGURE 5:**
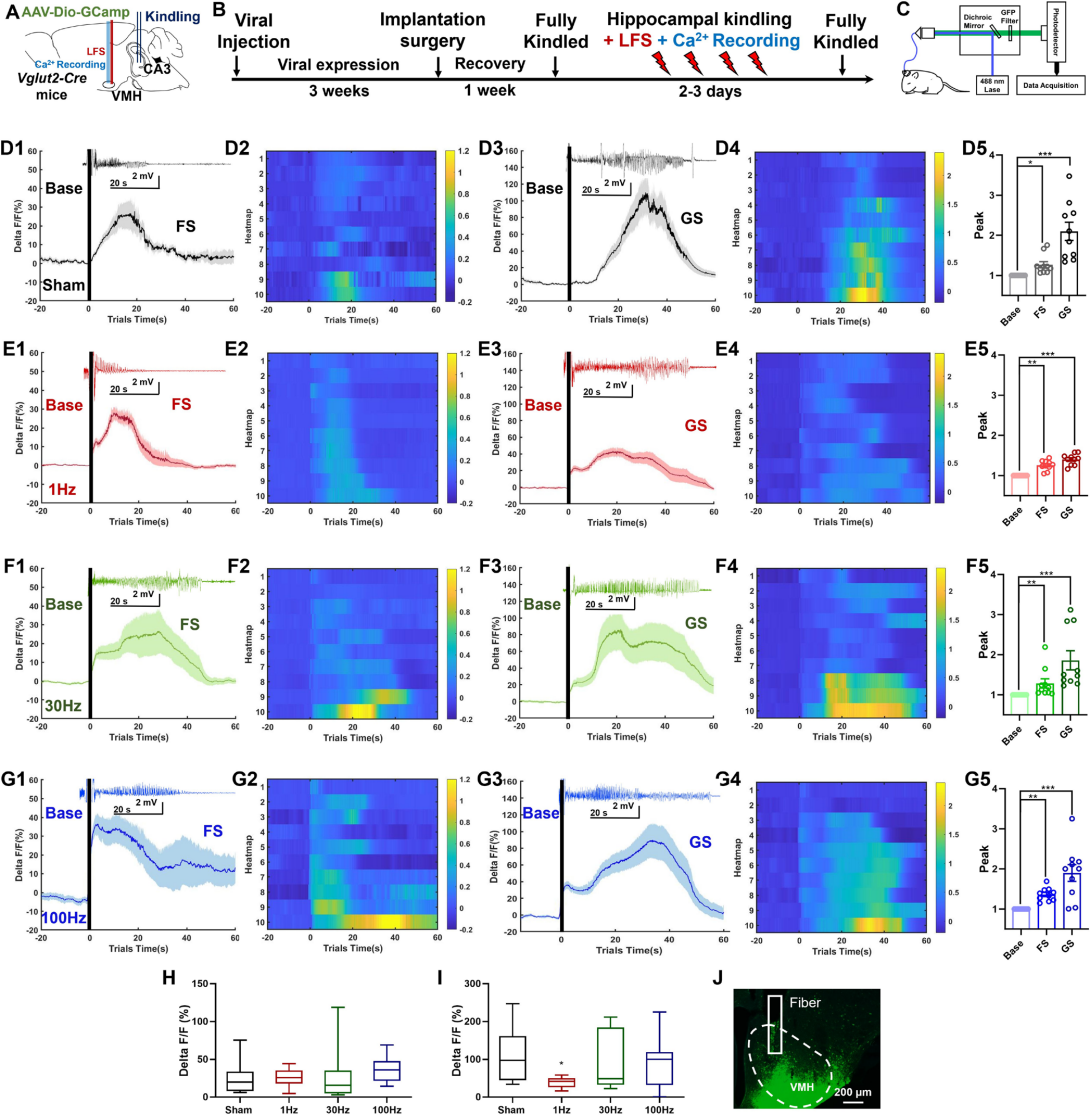
Low‐frequency stimulation attenuated the hyperactivities of glutamatergic neurons in the ventral medial hypothalamus during the kindling process. (A–C) The diagram of experiments for Ca^2+^ fiber photometry of VMH glutamatergic neurons in the hippocampal‐kindling model. The *AAV‐Dio‐GCamp* was injected into the VMH of Vglut2‐Cre mice (A). Then, the Ca^2+^ was recorded in each group during both FS and GS (B). The Ca^2+^ signals were elicited by a fluoresce excitor and recorded simultaneously (C). (D1‐E1) The average Ca^2+^ signals (*ΔF/F*) during FS in the sham (D1), 1 Hz (E1), 30 Hz (F1), and 100 Hz (G1) groups (upper panels are the representative seizure EEGs of each group). The corresponding heatmaps, in which each row represents one trial, were denoted in D2–G2. (D3–G3, D4–G4) The average Ca^2+^ signals (ΔF/F) and corresponding heatmaps during GS in the four groups (upper panels are the representative seizure EEGs of each group). (D5–E5) The comparison of the peak intensities of Ca^2+^ signals in mice at the baseline condition, and experiencing FS and GS. (H and I) In the comparisons of the peak intensities of Ca^2+^ signals during FS (H) and GS (I) among the four groups, mice experiencing VMH‐LFS exhibited the lowest intensity during GS. (J) The histological verification of GCaMP6s expression in coronal brain slices in the VMH. We performed 10 trials for each group. For D5‐G5, *p < 0.05, **p < 0.01, ***p < 0.001, one‐way ANOVA followed by Turkey's post hoc test. For I, *p < 0.05, compared with the sham group, nonparametric Kruskal–Wallis test was used.

Given that VMH^glu^ was the primary neuronal type in the VMH [35], and was significantly activated following seizures, we also want to know whether LFS influences the activities of VMH^glu^ by using optic fiber photometry in the hippocampus‐kindling model. First, we injected the AAV and implanted an optic fiber into the VMH of Vglut2‐Cre animals (Figure 5A). Then, hippocampal kindling was performed, followed by immediate VMH‐DBS. Each group's GCaMP6 fluorescence intensities were recorded during FS and GS in the kindling process (Figure 5B,C). Both FSs and GSs showed a considerable increase in calcium response (average signal *△F/F*) during hippocampal kindling, as expected (Figure 5D–G). Resulting in significantly higher peak intensities during both FS and GS in all groups (Figure 5D–G) as compared to the baseline circumstances. However, the elevated degrees appeared to be more significant in the sham and 30 and 100 Hz DBS groups (Figure 5D–G). To further confirm whether there exist differences in the peak calcium response among different groups, we then evaluated the peak calcium responses of the four groups under both FS and GS circumstances. Peak calcium responses in FS were equivalent to those in the sham group at 1, 30, and 100 Hz DBS (Figure 5H), whereas 1 Hz LFS considerably reduced peak calcium responses in GS (Figure 5I). After all the experiments, we postexamine the viral expression and fiber locations; as shown in Figure 5J, GCaMP6s was successfully expressed in the VMH, and the optic fiber was correctly inserted into the superficial VMH to ensure the recorded Ca^2+^ signals were from VMH^glu^. In brief, these results indicated that 1 Hz LFS effectively inhibited the activities of VMH^glu^ in the hippocampal‐kindling model. On the other hand, whether LFS would show an impact on physiological functions of VMH^glu^ was tested, and it was confirmed that VMH‐LFS would not significantly influence the calcium response in free‐moving *vglut2‐cre* mice which allowed full expression of *AAV‐Dio‐GCamp6s* in the VMH (Figure S4). To further explore the downstream circuitry basis of VMH^glu^ which can be modulated by inhibitory VMH‐LFS, anterograde neural tracing was performed via injecting *AAV‐Dio‐ArchT‐EYFP* into the VMH of *vglut2‐cre* mice (Figure S5). And we found that VMH^glu^ could send excitatory projections to multiple downstream regions ranging from the hippocampus, the thalamus, the midbrain, the brainstem, to the neocortical areas, suggesting the broad‐spectral antiseizure efficacy of VMH‐LFS maybe due to the inhibitory effects caused by LFS on wide‐projected VMH^glu^.

**FIGURE 6:**
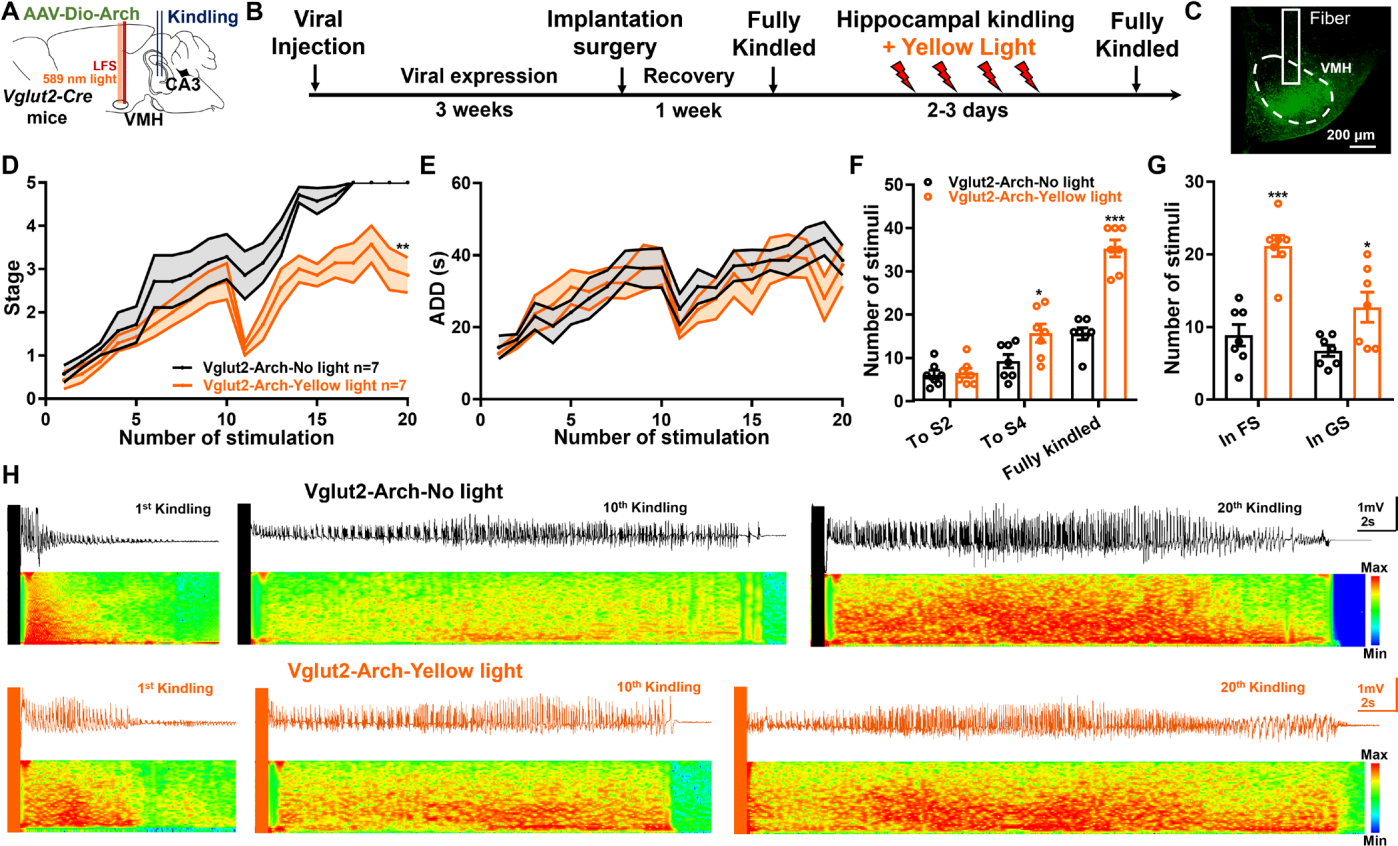
Selectively inhibition of the glutamatergic neurons in the ventral medial hypothalamus retards the hippocampal‐kindling process. (A and B) The diagram of experiments for optically inhibiting VMH glutamatergic neurons in the hippocampal‐kindling model. The *AAV‐Dio‐Arch* was injected into the VMH of Vglut2‐Cre mice, followed by the optic fiber implantation (A). After viruses were fully expressed, mice were applied to fast kindling procedures with a 30‐s‐long yellow‐light stimulation (DC, 589 nm) immediately after each kindling stimulation (B). (C) The histological verification of Arch expression in coronal brain slices in the VMH. (D and E) The progression of seizure stages (D) and ADDs (E) of both Vglut2‐Arch no‐light and Vglut2‐Arch yellow‐light groups. (F) The number of kindling stimulations needed to reach Stages 2 and 4 and fully kindled seizures. (G) The number of kindling stimulations that mice experienced to stay in FS (Stages 0–3) and GS (Stages 4 and 5). (H) The representative seizure EEGs and spectrums when receiving 1st, 10th, and 20th kindling stimulation for each group. Each group contains seven mice, for C, **p < 0.01, compared with the Vglut2‐Arch no‐light group; two‐way ANOVA. For F and G, *p < 0.05, *p < 0.001, compared with the Vglut2‐Arch no‐light group, unpaired *t* test.

Since LFS could inhibit the hyperactivities of VMH^glu^ during epileptic seizures, we further want to examine whether VMH^glu^ was responsible for its efficacy in the hippocampal‐kindling model via performing optogenetic experiments (Figure 6A). After full expression of the ArchT in VMH^glu^, the hippocampal‐kindling process was applied followed by yellow‐light stimulation to selectively inhibit VMH^glu^ (Figure 6B). After 4 weeks of viral expression, we confirmed Vlgut2‐Arch–positive cell bodies strictly located in the VMH (Figure 6C). As demonstrated in Figure 6D,E, optogenetic inhibition of VMH^glu^ effectively delayed the epileptogenesis process produced by kindling stimulation. This was further demonstrated in Figure 6F,G, where yellow‐light stimulation increased the number of stimulations required to achieve Stage 4 and fully kindled seizures (Figure 6F), as well as to remain in FS and GS during the kindling process (Figure 6G). Figure 6H shows representative seizure EEGs and spectrums. These results revealed that selectively suppressing VMH^glu^ matched the efficiency of LFS in the hippocampus‐kindling paradigm. Overall, the results showed that VMH^glu^ was linked with LFS's antiseizure efficacy.

## 4 Discussion

The selection of optimal DBS targets for epilepsy remains a major concern for neurologists. Current clinical trials suggest that the ATN, CMT, and hippocampus are evidence‐based effective targets for reducing seizures in refractory epilepsy [5, 7, 36, 37]. Although DBS at these three targets could achieve seizure reduction rates above 50%, it seems that DBS at these targets possesses therapeutic priorities for specific seizure types. CMT‐DBS was mainly effective for generalized‐onset seizures [7], ATN‐DBS showed priority in focal‐onset seizures originating from the temporal and frontal lobes [8], while patients with refractory TLE were suitable candidates for hippocampal DBS [10]. In this study, we provided direct evidence to demonstrate that VMH may be a potential DBS target with a broad antiseizure spectrum. As we discovered, VMH‐DBS effectively reduced the severity of both generalized and focal‐onset seizures across various epilepsy models. VMH‐DBS's broad‐spectral efficacy could be ascribed to its substantial participation in various types of seizures. Our *c‐fos* findings, which were consistent with earlier investigations, revealed that neurons in the VMH were engaged during both generalized and focal‐onset seizures [20, 21]. It has been revealed that crucial epilepsy‐related regions such as the ventral hippocampus, the amygdala, the cortices, the thalamus, and the brainstem were the main upstream regions of the VMH; these anatomical qualities may be the neural basis for VMH's widespread participation in seizures [38, 39, 40, 41]. Furthermore, we confirmed that VMH could send wide‐distributed projections to various brain areas ranging from the hippocampus, the thalamus, the midbrain, the brainstem, to the neocortical areas, perhaps this up‐/downstream neural circuitry basis lays the foundation of the broad‐spectral antiseizure efficacy of VMH‐LFS. Besides the neural circuitry basis, LFS at the VMH which belongs to the key component of the HPG axis may further lead to the neurochemical alteration of serotoninergic, cholinergic, and other wide‐distributed neural transmissions [42, 43]; these changes may be also responsible for its broad‐spectrum anti‐seizure efficacy. With the advantage of broad‐spectral antiseizure efficacy, our study further expanded the potential clinical use of VMH‐DBS.

Which parameter is suitable for DBS in epilepsy is still questionable. Based on the frequencies, DBS can be further divided into LFS and high‐frequency stimulation (HFS). With the benefits of safety and reversibility, LFS has gradually gained acceptance as the best parameter for managing seizures [44]. Here, in this study, we further demonstrated that only 1 Hz LFS was effective against various types of seizures. Unlike previous findings that VMH‐HFS was effective for obesity [45], we discovered that both 30 and 100 Hz DBS had minimal effects in most epilepsy models. This disparity could be attributed to the specific modulating effects of LFS on neuronal activity. Previous studies, including ours, have proposed that the delivery of recurring LFS treatments would induce long‐term depotentiation (LTD) efficacy in stimulated brain regions [46, 47]. This LTD would directly inhibit neuronal hyperexcitabilities via various mechanisms. It has been known that the epileptic seizures would cause the hyperactivity of the VMH (this is also the reason for the HPG axis irregularities in epilepsy). Here, we further revealed that LFS directly inhibited the hyperactivities of VMH^glu^, which is the main neuronal type in this region during the hippocampal‐kindling–induced epileptogenesis process. Inversely, we confirmed that VMH HFS (both 30 and 100 Hz) had no impact on the activities of VMH^glu^, this may be the reason why it is ineffective for seizures in most cases. Also, it has been found that VMH HFS would influence food intake, induce a systemic inflammatory response, and cause aggressive behaviors, according to previous clinical and laboratory evidence [48, 49, 50]. We proposed that 1 Hz LFS may be the ideal parameter for VMH‐DBS against epilepsy, with the advantages of broad‐spectral effectiveness and potentially less side effects, and these findings resonate with previous evidence that LFS may be better than HFS for intractable epilepsy [46].

Although epileptic seizures are often accompanied by psychopathological symptoms and HPG axis anomalies which are both dominated by the hypothalamus, the exact role of VMH in epilepsy is not sufficiently illustrated. Interestingly, we found that the main impact of VMH‐LFS on both generalized and focal‐onset seizures was to prolong the seizure latency. For generalized‐onset seizures induced by PTZ, VMH‐LFS effectively prolonged the latencies to each seizure stage, while for focal‐onset seizures, it only prolonged the latencies to the secondary‐generalized seizures. Based on this, we can deduce that VMH may partly possess seizure‐originating role in generalized‐onset seizures, while for focal‐onset seizures arising from cortical or hippocampal areas, VMH may participate in the seizure propagation. Also, previous findings about VMH in epilepsy lack cell‐type identification [20, 21]. Another early finding only confirmed the disruption of the epileptogenesis process in the flurothyl‐kindling model caused by lesioning the VMH [51]. In this study, we provided the first direct evidence that VMH^glu^ was a possible cellular target for epilepsy. As the *c‐fos*‐positive neurons in the VMH after hippocampal‐induced seizures were mainly glutamatergic, directly inhibiting VMH^glu^ by optogenetics mirrored the efficacy of LFS on the progression of seizure stages in hippocampal‐kindling model. Interestingly, we discovered that optogenetic inhibition of VMH^glu^ had no effect on ADDs, which is unique from LFS. This could be attributable to the different acting styles of the two tactics. As a proton pump, optically stimulating the Arch resulted in an instantaneous and precise hyperpolarization of the soma in Arch‐expressed neurons, while LFS possesses an extensive and long‐acting mode on the neurons via inducing LTD which was caused by synaptic transmissions. Thus, it can be deduced that the VMH^glu^ itself may mainly dominate the propagation of epileptic seizures. While the maintenance of seizures is more related to the neural transmission between the seizure‐originating zones and the VMH, we believe it is a highly promising approach for further demonstrating the unique functions of VMH in seizure propagation and maintenance.

However, we do admit that the current study still has some limitations. It is still unillustrated whether the neural circuitry basis of VMD‐LFS was identical for different types of seizures. Also, previous studies have confirmed the dorsal and ventral parts of VMH were functionally heterogeneous in the physiological conditions [52], the efficacies of LFS at the different subregions of VMH for seizures need further investigation.

## 5 Conclusion

To sum up, in this study, we proposed preclinical evidence to demonstrate the VMH as an ideal target for controlling seizures across epilepsy models, and VMH‐LFS possesses a broad‐spectral antiseizure efficacy via inhibiting hyperactivities of VMH^glu^.

## Ethics Statement

All the experiments were approved by the guidelines of the Animal Advisory Committee of Zhejiang Chinese Medical University (Approved Number: 20230306‐10).

## Conflicts of Interest

The authors Zhong Chen is a member of the editorial board of *CNS Neuroscience & Therapeutics*.

## Supporting information


Figure S1.
Figure S2.
Figure S3.
Figure S4.
Figure S5.

## Data Availability

The data that support the findings of this study are available from the corresponding authors upon reasonable request.
