# Supplementary material for: Low‐Frequency Stimulation at the Ventromedial Hypothalamus Exhibits Broad‐Spectrum Efficacy Across Models of Epilepsy
Source: CNS Neurosci Ther. 2025 Feb 9;31(2):e70265. doi: 10.1111/cns.70265 (PMC11808192; doi:10.1111/cns.70265)
Supplement: Supplementary file 1 — Figure S1. Figure S2. Figure S3. Figure S4. Figure S5. [file CNS-31-e70265-s001.docx]

**Submitted to *CNS Neuroscience & Therapeutics***

***Supplementary Materials***

**Low Frequency Stimulation at the** **Ventromedial Hypothalamus Exhibits** **Broad Spectrum Efficacy Across Models of Epilepsy**

Shuang Zou^1,*^, MD, Yiwei Gong^1,*^, PhD, Mengqi Yan^1,*^, MS, Zhijian Yuan^1,*^, MS, Minjuan Sun^1^, PhD, Shuo Zhang^1,2^, MS, Yuanzhi Yang^1^, PhD, Xiongfeng Guo^1^, MS, Lan Huang^1^, MS, Fan Fei^1^, PhD, Yi Wang^1^, PhD, Zhong Chen^1,#^, PhD, Cenglin Xu^1,#^, PhD

From the:

^1^ Key Laboratory of Neuropharmacology and Translational Medicine of Zhejiang Province, The Second Affiliated Hospital of Zhejiang Chinese Medical University (Zhejiang Xinhua Hospital), School of Pharmaceutical Sciences, Zhejiang Chinese Medical University, Hangzhou, China.

^2^ Department of Pharmacy, The First Affiliated Hospital of Zhejiang Chinese Medical University (Zhejiang Provincial Hospital of Chinese Medicine), Hangzhou, China.

^*^These authors contributed equally

^#^Corresponding author:

Prof. Cenglin Xu. Tel & Fax: +86-571-86618319. E-mail: [xucenglin5zz@zju.edu.cn](mailto:xucenglin5zz@zju.edu.cn)

Prof. Zhong Chen. Tel & Fax: +86-571-86618660. E-mail: [chenzhong@zju.edu.cn](mailto:chenzhong@zju.edu.cn)

**Supplementary Figure Legends**

**
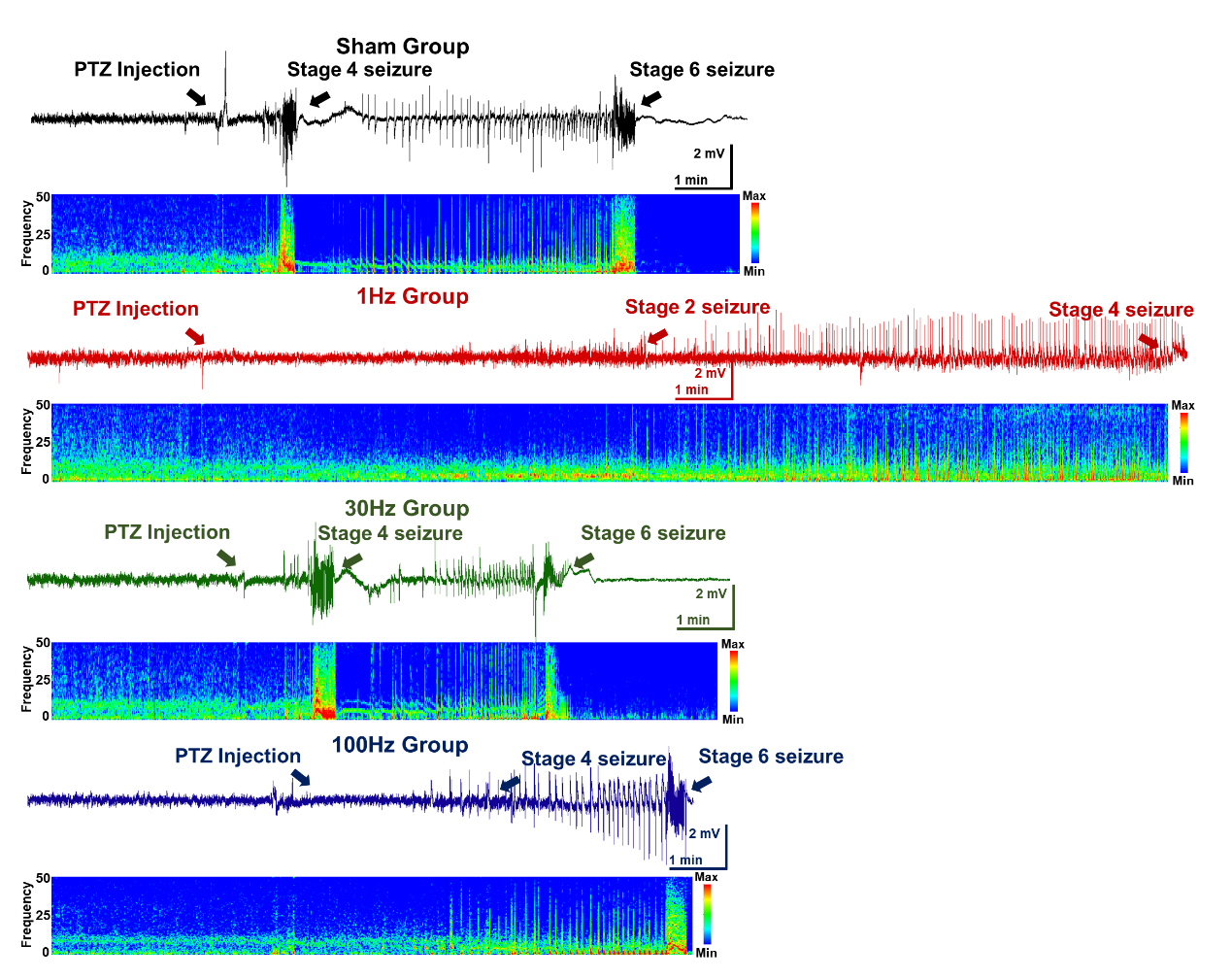
**

**Supplementary Figure 1. The representative seizure EEGs and spectrums in the 100 mg/kg PTZ induced acute seizure models.** The seizure EEGs and spectrums of the sham (black), 1 Hz (red), 30 Hz (green), and 100 Hz (blue) groups were showed. The arrows denoted PTZ injection, representative stage 4 and 6 seizures.


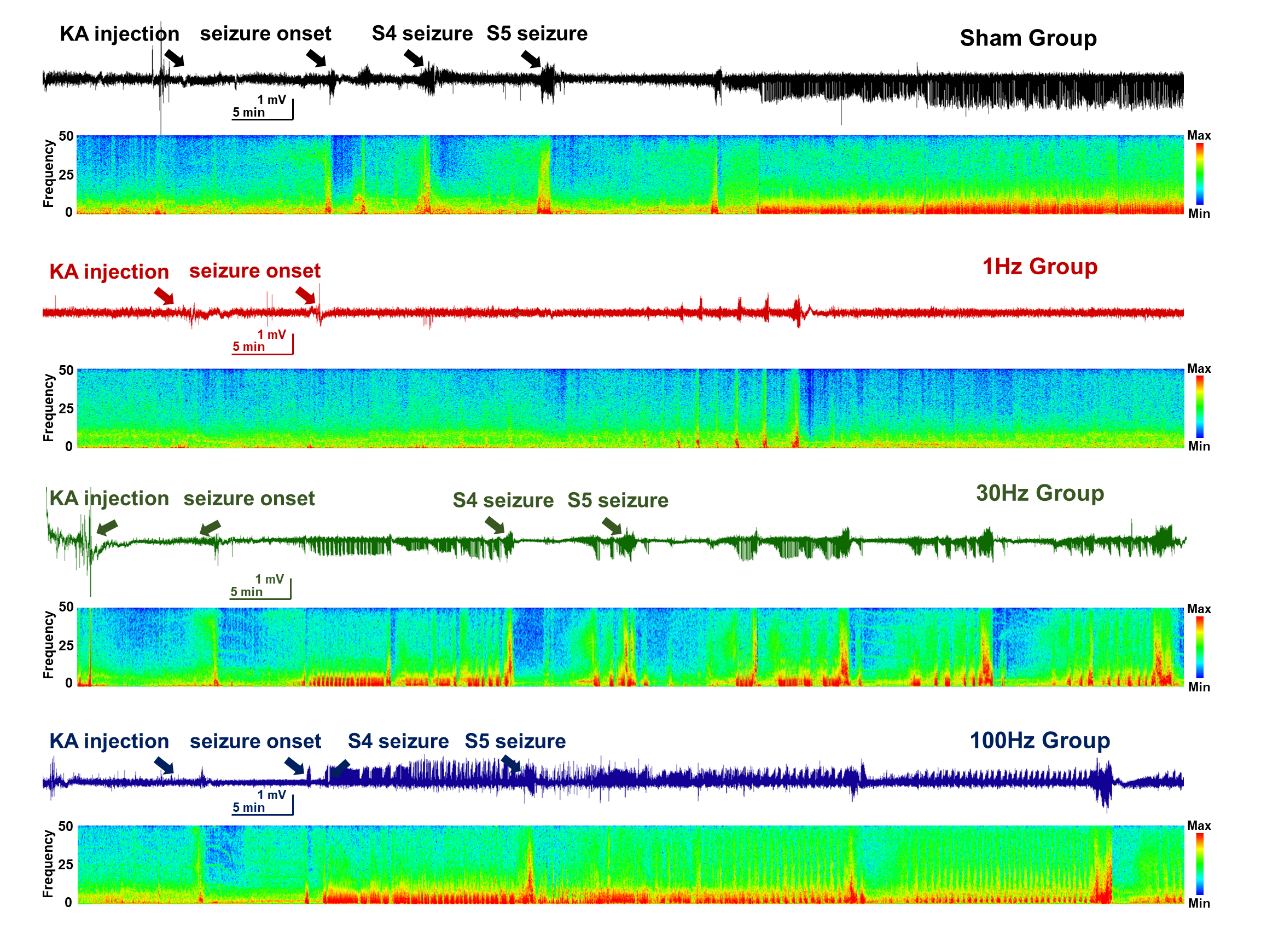


**Supplementary Figure 2. The representative seizure EEGs and spectrums in the acute cortical KA models.** The seizure EEGs and spectrums of the sham (black), 1 Hz (red), 30 Hz (green), and 100 Hz (blue) groups were showed. The arrows denoted the KA injection, the seizure onset, and the representative stage 4 and 5 seizures.


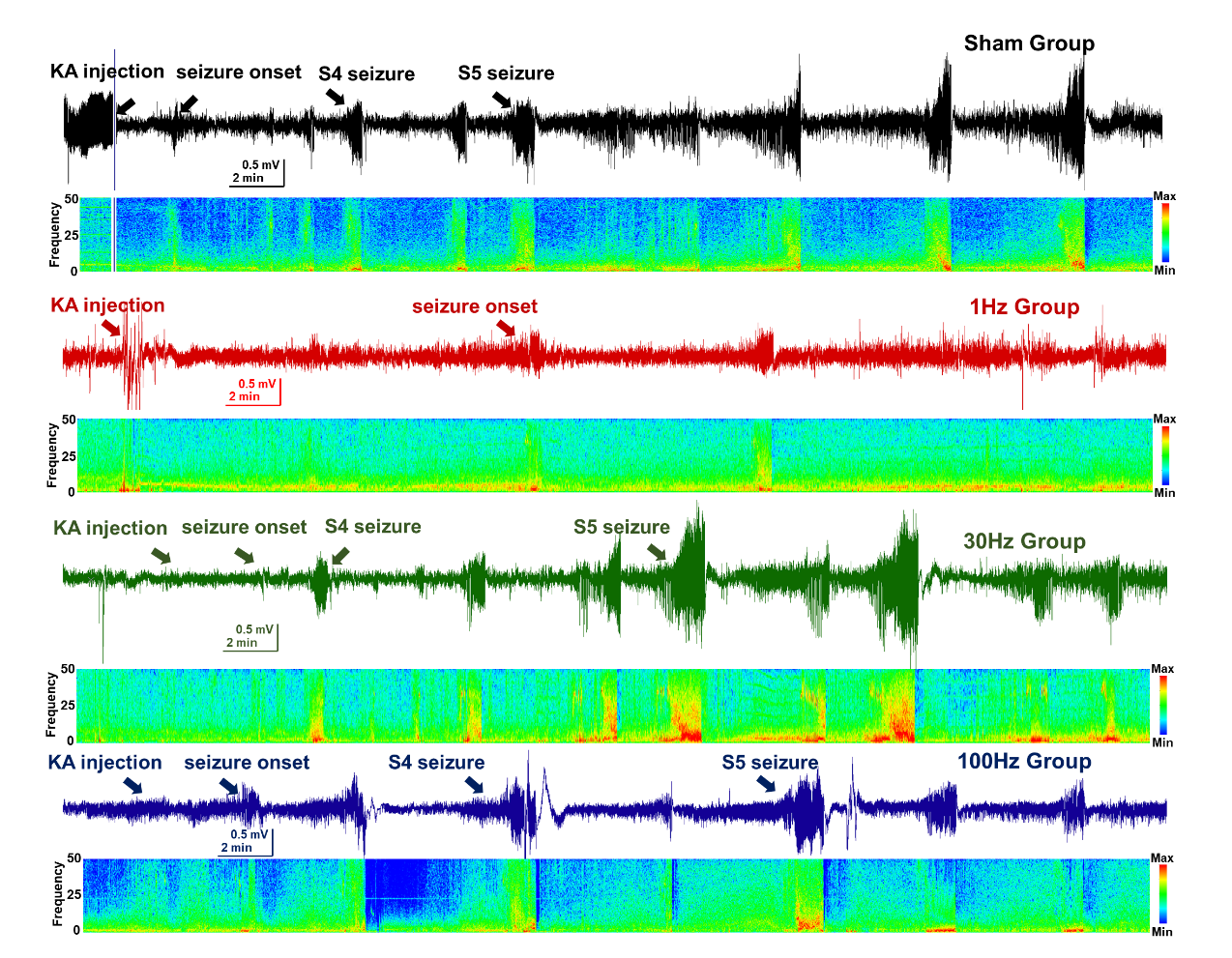


**Supplementary Figure 3. The representative seizure EEGs and spectrums in the acute hippocampal KA models.** The seizure EEGs and spectrums of the sham (black), 1 Hz (red), 30 Hz (green), and 100 Hz (blue) groups were showed. The arrows denoted the KA injection, the seizure onset, and the representative stage 4 and 5 seizures.


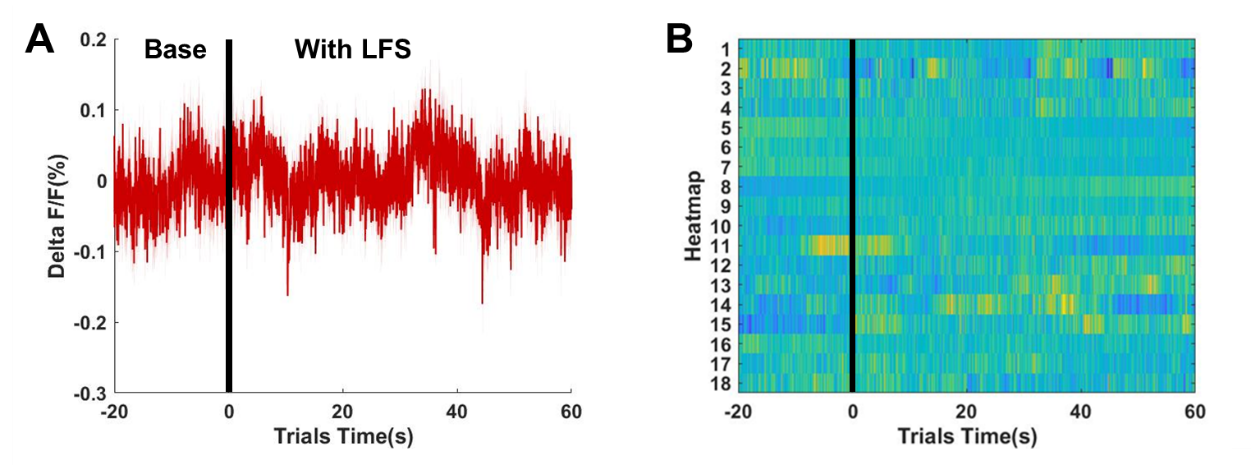


**Supplementary Figure 4. LFS did not influence the calcium activities of glutamatergic neurons of the VMH in free-moving mice.** The *AAV-Dio-GCamp* was injected into the VMH of *Vglut2-Cre* mice, when fully expressed, the Ca^2+^ signals were recorded in free-moving mice before and during applying LFS at the VMH. (A) The average Ca^2+^ signals (*ΔF/F*) before (base) and during LFS treatment (with LFS) in free-moving mice. The corresponding heatmaps, in which each row represents one trial were denoted in (B).


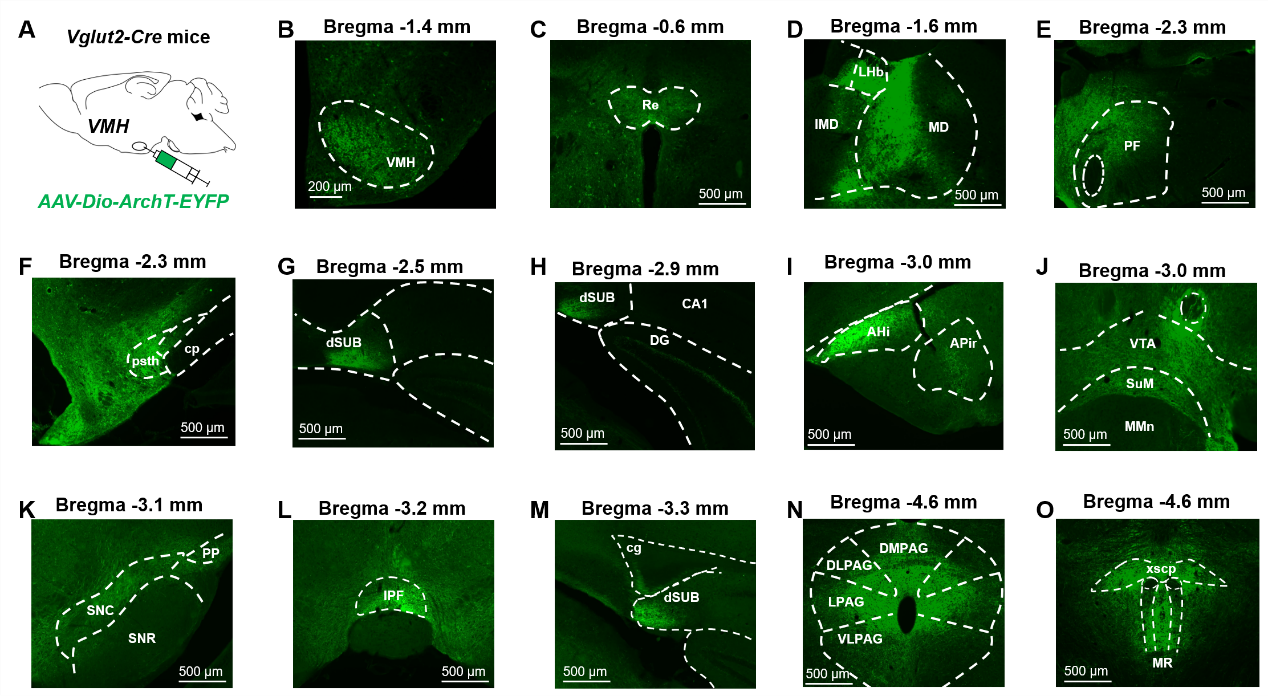


**Supplementary Figure 5. The anterograde viral tracing of the glutamatergic neurons of the VMH.** (A) The *AAV-Dio-ArchT-EYFP* was injected into the VMH of *Vglut2-cre* mice to allow the expression of fluorescent EYFP on the projected dendrites in the downstream areas. (B-O) Primary outputs of the glutamatergic neurons in the VMH. Series of coronal sections from a representative mouse showing the major outputs of the VMH. Coronal brain slices were showed in order from the anterior to the posterior regions. VMH, ventromedial hypothalamus. Re, reuniens thalamic nucleus. LHb, lateral habenular nucleus. IMD, intermediodorsal thalamic nucleus. MD, mediodorsal thalamic nucleus. PF, parafascicular thalamic nucleus. PSTh, parasubthalamic nucleus. CP, cerebral peduncle. dSUB, dorsal subiculum. DG, dentate gyrus. AHi, amygdalohippocampal area. APir, amygdalopiriform transition area. VTA, ventral tegmental area. SuM, supramammillary nucleus. MMn, medial mammillary nucleus. SNC, substantia nigra, compact part. SNR, substantia nigra, reticular part. PP, peripeduncular nucleus. IPF, interpeduncular fossa. Cg, cingulum. PAG, periaqueductal gray. MR, medial raphe. Xscp, decussation of the superior cerebellar peduncle.
